# When HIV Immunodeficiency and Heterochromia Confuse the Issue: Recurrent Zoster Uveitis Mistaken for Fuchs’ Uveitis

**DOI:** 10.18502/jovr.v16i2.9094

**Published:** 2021-04-29

**Authors:** Ioannis Papasavvas, Bruno Jeannin, Carle Pierre Herbort

**Affiliations:** Retinal and Inflammatory Eye Diseases, Centre for Ophthalmic Specialized Care (COS), Clinic Montchoisi Teaching Centre, Lausanne, Switzerland

**Keywords:** Herpes Zoster Uveitis, Heterochromia, HIV

## Abstract

**Purpose:**

We report a case with iris heterochromia misdiagnosed as Fuchs' uveitis which finally turned out to be a unilateral zoster uveitis in an HIV-positive patient.

**Case Report:**

A 45-year old patient was seen for a recurrent right anterior uveitis treated with prednisolone 1% drops BID. The iris of the right eye was hypochromic and atrophic and several small granulomatous keratic precipitates (KPs) were present. After discontinuation of corticosteroid drops, severe uveitis developed with mutton-fat KPs, and laser flare photometry (LFP) increased from 20 to 50.3 ph/ms. He had presented with right zoster ophthalmicus two years earlier and HIV-serology revealed to be positive.

**Conclusion:**

Iris heterochromia is not a good disease-defining criterion for Fuch's uveitis even when typical KPs are present and can lead to misdiagnosis. More reliable criteria including stellate KPs, low LFP values, absence of synechiae, vitreitis, and disc hyperfluorescence, all absent in this case, should be sought to confirm or exclude the diagnosis.

##  INTRODUCTION 

Fuchs' uveitis (FU) is often underdiagnosed because too much importance is given to iris heterochromia at the expense of other more disease-defining clinical signs. The disease was described by Ernst Fuchs at the beginning of the last century in Vienna, Austria, inhabited by a Caucasian population.^[1,2]^ Iris heterochromia had a prominent part in the definition of the disease in his publications^[1]^ and his textbook, which was translated into more than 10 languages including countries with all brown iris populations. In these countries such as Japan^[3]^ and many others, FU was underdiagnosed because clinicians were (1) looking in vain for heterochromia that does not exist in brown irises and (2) ignored the clinical sign of vitreitis present in close to 100% of cases.^[4]^ Therefore, strong and universal disease-defining signs have to be used to confirm or exclude FU. These criteria, all present in >90% of non-operated cases, include vitritis,^[4]^ small stellate keratic precipitates (KPs), low aqueous flare values measured by laser flare photometry (LFP, ≤ 20 ph/ms),^[5]^ absence of posterior synechiae, difference of texture of irises between the two eyes, absence of cystoid macular oedema, and hyperfluorescent disc on fluorescein angiography (FA).^[6]^ Here, we present a case where iris heterochromia led to falsely diagnosed FU in a case of recurrent zoster uveitis in an immunocompromised patient.

##  CASE REPORT

A 45-year old teacher was referred to our uveitis clinic by his treating eye doctor for a recurrent right granulomatous uveitis. History revealed that he had gone through right herpes zoster ophthalmicus (HZ.O) two years prior.

At presentation, the patient was under the treatment of 1% prednisolone acetate eye drop, twice daily. Uncorrected visual acuity ODS was 1.0 on the Snellen chart.

**Figure 1:**
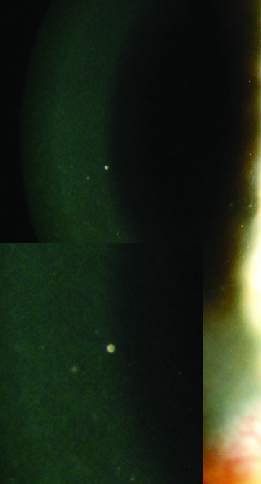
Sparse micro-granulomatous KPs, seen in magnification (insert) at presentation under the treatment of 1% prednisolone drops BID.

**Figure 2:**
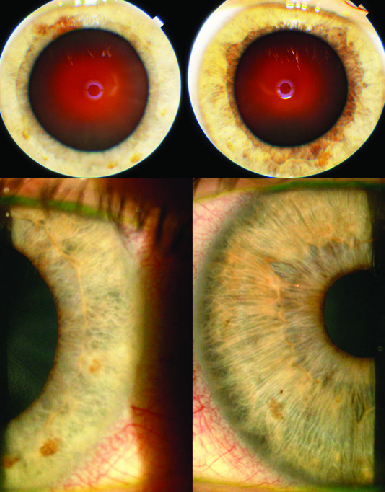
Heterochromia with a lighter-colored iris on the right (top). Bottom two pictures show a right discolored iris with an altered hyalinized iris texture (left picture).

**Figure 3:**
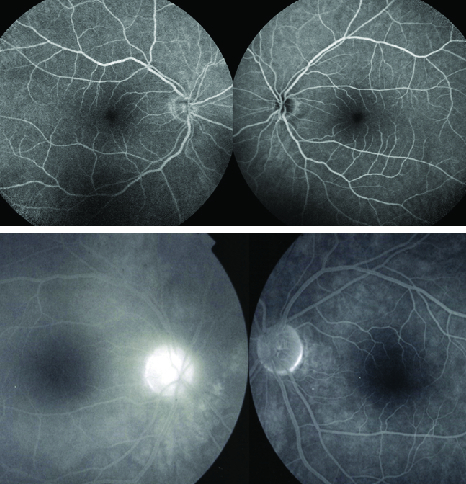
Absence of disc hyperfluorescence in the right affected eye on FA in the presented case (top angiographic frames). In comparison, bottom two frames show the typical disc hyperfluorescence seen in Fuchs' uveitis.

**Figure 4:**
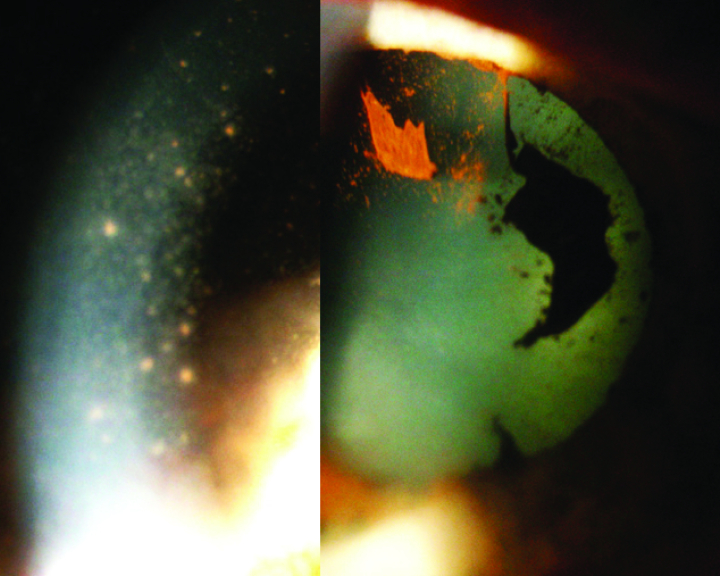
Massive mutton-fat KPs deposition on the endothelium in the right eye (left picture) after discontinuation of corticosteroid drops and posterior ruptured synechiae (right picture), incompatible with Fuchs' uveitis.

On the right corneal endothelium, there were rare small randomly distributed stellate KPs compatible with FU [Figure 1]. The anterior chamber was uninflamed on slit-lamp examination but LFP measured a sub-clinical inflammation of 20.2 ph/ms (normal = 3–6 ph/ms). Intraocular pressure was 12 mmHg OD and 14 mmHg OS. An iris heterochromia was noted with a lighter iris on the right and an altered hyalinized iris surface [Figure 2]. There was no sectorial iris atrophy. There were rare cells in the anterior vitreous OD. Fundus examination as well as retinal and choroidal optical tomography (OCT) were normal. We made the diagnosis of FU based on typical KPs and heterochromia and stopped the corticosteroid drops. FA performed the following day did not show the disc hyperfluorescence usually seen in FU [Figure 3] nor the limited peripheral vascular leakage often present in FU. Ten days later, the patient returned with a severe granulomatous uveitis with numerous mutton-fat KPs [Figure 4] and increase of LFP values to 51.4 ph/ms associated with posterior synechiae [Figure 4]. The diagnosis was revised to recurrent zoster uveitis and the patient was treated with valacyclovir 1000 mg TID and 1% prednisolone acetate drops 5× per day. The uveitis responded well to treatment with resolution of the mutton-fat KPs and reduction of LFP values to 20.7 ph/ms. However, at each tapering attempt, the uveitis recurred. As we suspected an immune deficiency to varicella-zoster virus or a more general immunodepression, we asked the patient to give us his consent to search for HIV infection. HIV serology was positive, and the CD4 lymphocyte count was reduced to 332 cells/mm3. After the two attempts to taper the treatment, we decided to leave the patient under combined systemic antiviral treatment and corticosteroid drops and to wait until the CD4 count would be back to normal after the initiation of antiretroviral therapy.

##  DISCUSSION

Iris heterochromia is still too strongly associated with FU while better disease-defining criteria have been put forward.^[6,7]^ Outside FU, viral-related iris heterochromia has been described in association with cytomegalovirus^[8]^ and with Ebola virus.^[9]^ In a large series of viral anterior uveitis cases with etiologic agents proven by aqueous PCR, iris heterochromia was never detected in varicella-zoster uveitis.^[10]^ Although a thorough literature search remained negative, it is not impossible “a priori” that zoster uveitis can produce heterochromia, which was the case in our patient.

The differential diagnosis of FU and herpetic anterior uveitis can be easier when large, mutton fat-like KP's are present, which can exclude FU. In the early stages of herpetic anterior uveitis, mutton-fat KP's can very rarely be absent, confusing the ophthalmologist. They can also be absent after a period of local corticosteroid treatment, as it happened in our case, which can lead to misdiagnosis. Discontinuing the corticosteroid drops and re-examining our patient after 10 days revealed abundant mutton-fat KPs, increase of flare and posterior synechiae, features incompatible with FU.

Once FU was excluded and after two failed attempts to taper antiviral and corticosteroid treatment, we decided to search for an immune deficiency specifically to varicella-zoster virus (VZV) or a more global immunodeficiency. It is known that herpes zoster in patients under the age of 50 is associated with HIV infection. In patients with a CD4 count from 200 to 349 cells/mm${}^{3}$ which was the case for our patient, herpes zoster is listed as the third most frequent clinical condition (12%) of missed opportunity for earlier diagnosis of HIV infection.^[11]^ Another trial showed that herpes zoster was a clinical event in late presenters with HIV infection indicating missed opportunities for earlier diagnosis in 19.8% of the cases.^[12]^

On the other hand, it was shown in a series of 89/581 (14.3%) HIV-positive uveitis patients that the first cause of anterior uveitis was due to VZV amounting to 43%.^[13]^

VZV was shown to persist for much longer in tissues of HIV-positive patients and even that there was a rebound inflammation when anti-retroviral treatment was started.^[14,15]^ Therefore, we left the patient under dual systemic antiviral and topical corticosteroid therapy after introduction of anti-retroviral therapy and CD4 count recovery.

The ophthalmologist, in presence of VZV pathology in patients under the age of 50, should function as a whistle blower in order not to miss an early diagnostic opportunity for HIV infection.

##  Financial Support and Sponsorship

Nil.

##  Conflicts of Interest

None.
